# Advance Care Planning Among Healthcare Professionals During COVID-19

**DOI:** 10.1093/geroni/igab046.3660

**Published:** 2021-12-17

**Authors:** Jessica Hahne, Brian Carpenter

**Affiliations:** 1 Washington University in St. Louis, St. Louis, Missouri, United States; 2 Washington University in St. Louis, Saint Louis, Missouri, United States

## Abstract

The COVID-19 pandemic is bringing healthcare professionals face to face with gravely ill patients in complex clinical situations. Caring for patients experiencing lengthy intubation, heavy sedation, rapid decline, and significant distress at the end of life has the potential to shift the perspectives of healthcare professionals regarding their own end-of-life care. This study explored advance care planning (ACP) among medical professionals and whether COVID-19 experiences altered their healthcare preferences and planning. Ninety-eight professionals (mean age = 45.6, 75% female) completed an online survey about ACP conversations, behavioral intentions to pursue ACP, openness to life-prolonging interventions, and ACP resource needs. ACP conversations were most extensive with spouse/partner (89% had talked about care preferences “some” or “a lot”) and to a lesser extent with parents (64%) and other healthcare providers (69%). Two-thirds (67%) of respondents had an ACP conversation since the start of COVID. Among respondents who had not completed ACP documents, 64% had taken some step toward ACP. When asked whether their preferences for life-prolonging medical interventions had changed, 70% reported no change, 16% reported being less open, and 14% reported being more open. A majority (60%) requested resources to help them pursue ACP on their own, although many (42%) were interested in assistance at their workplace. Given that only 37% of our sample had themselves completed an advance directive, our results suggest now may be a critical moment to engage professionals in ACP, considering how their experience during the pandemic has motivated ACP conversations and a reconsideration of preferences.

